# High-resolution far-field ghost imaging via sparsity constraint

**DOI:** 10.1038/srep09280

**Published:** 2015-03-19

**Authors:** Wenlin Gong, Shensheng Han

**Affiliations:** 1 Key Laboratory for Quantum Optics and Center for Cold Atom Physics of CAS, Shanghai Institute of Optics and Fine Mechanics, Chinese Academy of Sciences, Shanghai, 201800, China

## Abstract

Ghost imaging (GI) is a method to nonlocally image an object with a single-pixel detector. However, the speckle's transverse size at the object plane limits the system's imaging resolution for conventional GI linear reconstruction algorithm. By combining the sparsity constraint of imaging object with ghost imaging method, we demonstrate experimentally that ghost imaging via sparsity constraint (GISC) can dramatically enhance the imaging resolution even using the random measurements far below the Nyquist limit. The image reconstruction algorithm of GISC is based on compressive sensing. Factors affecting the reconstruction quality of high-resolution GISC, such as the receiving system's numerical aperture and the object's sparse representation basis, are also investigated experimentally. This high-resolution imaging technique will have great applications in the microscopy and remote-sensing areas.

Far-field high-resolution imaging is always an important topic in imaging science. In practical applications, the imaging resolution is mainly restricted by the system's Rayleigh limit and detection signal-to-noise ratio (SNR)^1^,^2^. For example, the telescope with a large aperture is currently very difficult to be manufactured, thus the imaging resolution is basically circumscribed with the optical system's Rayleigh limit in remote sensing. For fluorescence imaging, because the fluorescent is weak and easy to be disturbed by the stray light in detection, the imaging resolution is limited mainly by the detection SNR.

Over the last decades, numerous ‘hardware' methods have been invented to improve the resolution of far-field imaging. Several techniques based on point-by-point scanning or fluorescence imaging have been introduced to improve the imaging resolution^3^,^4^,^5^,^6^,^7^. However, they require scanning or repetitive experiments, which limits real-time applications and makes them impossible to be applied in the field of imaging such as remote sensing. Apart from hardware solutions, several algorithmic approaches for far-field high-resolution imaging have been suggested by using additional a priori information on the optical system^8^,^9^,^10^,^11^,^12^. However, the degree of improvement is extremely sensitive to both noise in the measured data and the accuracy of the assumed a priori knowledge^2^,^8^,^9^,^10^,^11^,^12^. In addition, for an *N*-pixel image, these high-resolution imaging methods require at least *N* samples to reconstruct the image (this is called the Nyquist limit of the measurement).

Ghost imaging (GI), which is based on the quantum or classical correlation of fluctuating light fields, has demonstrated theoretically and experimentally that one can nonlocally image an unknown object without scanning the object, by using a single-pixel detector at the object path^13^,^14^,^15^,^16^,^17^,^18^,^19^,^20^. Because all the photons reflected (or transmitted) from the object illuminate the same single-pixel detector, this technique has the ability of high detection SNR. However, the imaging resolution of this technique is limited by the speckle's transverse size at the object plane for conventional GI linear reconstruction algorithm^16^,^17^. When signals satisfied a certain sparsity constraint, Donoho had demonstrated mathematically that super-resolution restoration was possible^21^,^22^. Recently, the image's sparsity has been taken as a quite general assumption, a compressive sensing (CS) technique enables the reconstruction of an *N*-pixel image from much fewer than *N* global random measurements^23^,^24^. This technique has already been successfully applied to super-resolution imaging^25^,^26^, remote sensing^27^,^28^, and compressive imaging^29^,^30^,^31^. For GI, the fluctuating light field obeys Gaussian statistical distribution and the measurement process is globally random. Therefore, when CS is applied to the image reconstruction of GI, high-resolution far-field ghost imaging via sparsity constraint (GISC) is possible with the use of random measurement below Nyquist limit because a natural object can be sparsely expressed in a proper representation basis (or under a suitable basis transform)^23^,^24^.

In this paper, we have experimentally demonstrated the high-resolution ability of GISC, by comparing the reconstruction results of GI and GISC techniques. We also discuss the effect of receiving system's numerical aperture and the object's sparse representation basis on the quality of high-resolution GISC.

## Results

### Experimental setup

**Figure 1:**
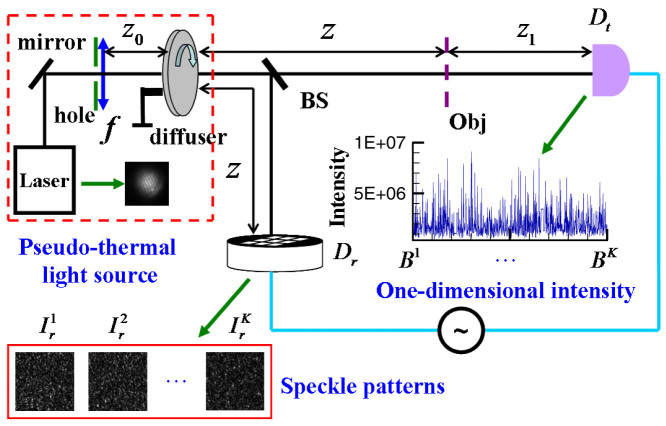
The experimental schematic of lensless far-field ghost imaging with pseudo-thermal light.

**Figure 2:**
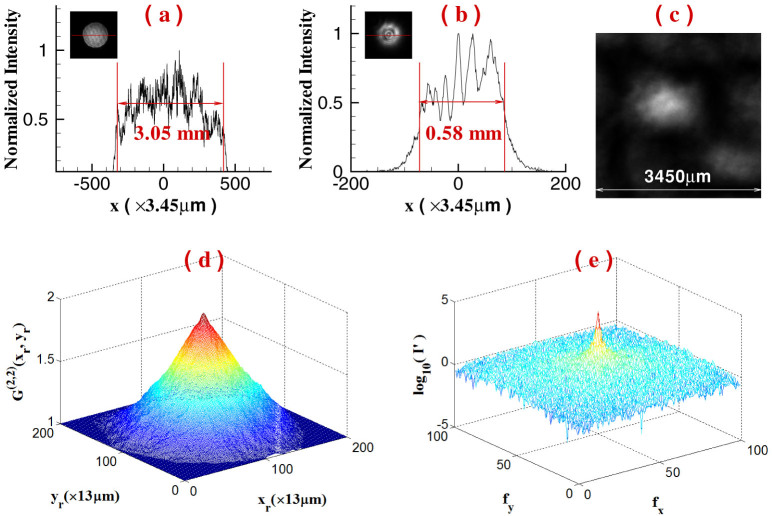
The characters of some important parameters used in the experiment. (a). The profile of the laser beam through the hole before the lens *f* and its cross-section at maximum value (along the red-line direction of the image); (b). the profile of the laser beam on the diffuser and its cross-section at maximum value (along the red-line direction of the image); (c). an image of a single speckle pattern record by the CCD camera *D_r_*; (d). the normalized second-order correlation distribution of light field at the reference detection plane; (e). the Fourier-transform distribution of (d).

Fig. 1 presents experimental schematic of lensless far-field GI with pseudo-thermal light. The scheme is similar to standard pseudo-thermal GI two-detectors setup mentioned in Ref. 30, but the speckle's transverse size at the object plane is too large to resolve the object and the test detector is fixed in the far field of the object, thus a single pointlike detector is enough to record global information from the object. In the experiment, as shown in Fig. 1, a Gaussian-shape laser (the wavelength λ = 650 nm and the diameter 5.0 mm) firstly goes through a hole (the diameter about 3.05 mm, see Fig. 2(a)) and then is focused onto a diffuser by a lens with the focal length *f* = 250 mm. The distance between the lens *f* and the diffuser is about *z*_0_ = 190 mm and the beam diameter on the diffuser is about D = 0.58 mm (see Fig. 2(b)). When the diffuser is slowly rotating, a pseudo-thermal light source can be generated^16^,^17^,^18^. Next, the light modulated by the diffuser is divided by a beam splitter (BS) into a test and a reference paths. In the test path, the light goes through a double-slit (slit width a = 100 *μ*m, slit height h = 500 *μ*m and center-to-center separation d = 200 *μ*m) and then to a detector *D_t_* fixed in the far field of the object (namely 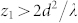). In the reference path, the same light propagates directly to a charge-coupled device (CCD) camera *D_r_*. Both the object and the CCD camera *D_r_* are located in the far field of the source (namely 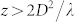). In addition, the reconstruction algorithms used for GI and GISC are the same as in Ref. 30.

### Experimental results

The parameters listed in Fig. 1 are set as follows: *z* = 1200 mm, the pixel size of the camera *D_r_* is 13 *μ*m × 13 *μ*m, and the single-shot exposure time is set to 1 ms. Fig. 2(c) presents an image of a single speckle pattern measured by the camera and the normalized second-order correlation distribution of light field at the reference detection plane is displayed in Fig. 2(d)^19^. For GI, the resolution limitation is determined by the full-width at half-max of normalized second-order correlation distribution, which is also equal to the transverse size of the speckle shown in Fig. 2(c)^16^,^17^. By operating the Fourier transform to the normalized second-order correlation distribution, the angular spectrum illuminating the object is shown in Fig. 2(e).

**Figure 3:**
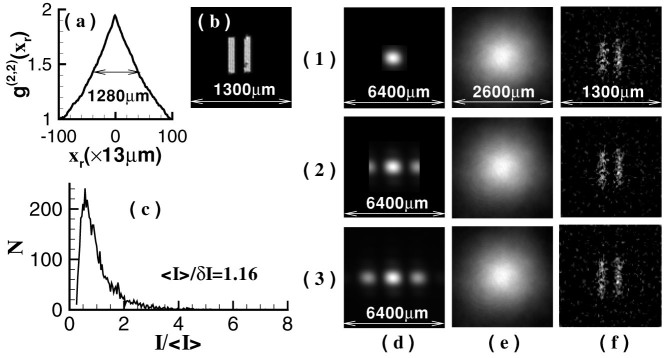
Experimental reconstruction of a double-slit in different receiving areas with *z*_1_ = 500 mm (the speckle's transverse size at the object plane Δ*x_s_* = 1280 mm). (a). The cross-section curve of normalized second-order correlation distribution at (x_r_, y_r_ = 100) direction displayed in Fig. 2(d) (the curve's full-width at half-max is corresponding to the resolution limitation of GI); (b). the object (100 × 100 pixels, the pixel size is 13 *μ*m × 13 *μ*m); (c). the probability distribution of the intensity values measured by the bucket detector *D_t_*relative to the statistical mean; (d). the object's Fourier-transform diffraction patterns received by the test detector *D_t_*; (e). GI reconstruction results (*K* = 10000); (f) GISC reconstruction results (with *K* = 3000 (*K*/*N* = 30% the Nyquist limit)). The receiving areas of the detector *D_t_* shown in (1–3) are 1.6 mm × 1.6 mm, 3.2 mm × 3.2 mm, and 6.4 mm × 6.4 mm, respectively.

**Figure 4:**
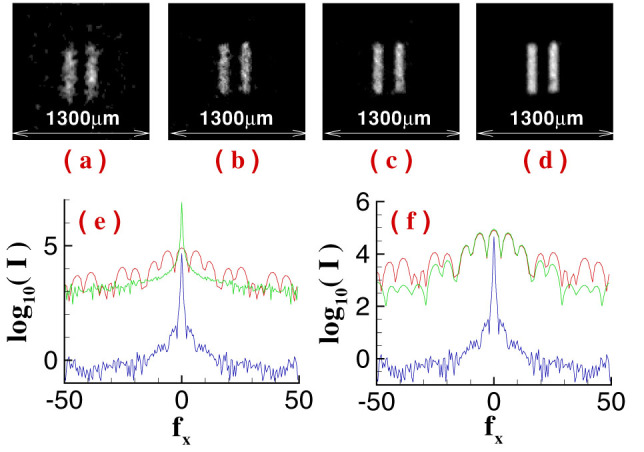
Experimental GISC reconstruction results of the same double-slit in different distances *z*_1_, and the other conditions are the same as Fig. 3 (*K* = 4000, namely 40% the Nyquist limit). (a). *z*_1_ = 500 mm; (b). *z*_1_ = 200 mm; (c). *z*_1_ = 100 mm; (d) *z*_1_ = 10 mm. The receiving area of the detector *D_t_* is 6.4 mm × 6.4 mm. The green solid curves displayed in (e) and (f) are the cross-section of Fourier-transform distributions of GI and GISC reconstruction results at (f_x_, 0) direction in the case of (d), respectively. The red solid curve is the cross-section of Fourier-transform distribution of the object shown in Fig. 3(b) at (f_x_, 0) direction. The blue solid curve is the cross-section of the image shown in Fig. 2(e) at (f_x_, 0) direction.

To demonstrate the high-resolution ability of GISC, Fig. 3 and Fig. 4 present experimental results of a double-slit recovered by GI and GISC methods in different receiving areas *L*_1_ × *L*_1_ and different distances *z*_1_, using the schematic shown in Fig. 1. For GISC method, we have utilized the gradient projection for sparse reconstruction algorithm^32^ and the double-slit is sparsely expanded in Cartesian representation basis. By measuring the normalized second-order correlation distribution displayed in Fig. 2(d), as shown in Fig. 3(a), its cross-section curve's full-width at half-max is about 1280 *μ*m, which coincides with the theoretical result Δ*x_s_* ≈ *λz*/*D* = 1345 *μ*m^16^,^17^. Therefore, as shown in Fig. 3(e), the object's image can not be reconstructed by conventional GI linear reconstruction algorithm because the speckle's transverse size at the object plane Δ*x_s_* is much larger than center-to-center separation of the object^16^,^17^. However, the imaging resolution can be dramatically improved by GISC method even if the number of random measurements used for image recovery is far below the Nyquist limit (see Fig. 3(f) and Fig. 4(a–d)). Usually, similar to the k-space spectral analysis method^34^, the improvement degree of imaging resolution can also be evaluated by measuring the angular spectrum of reconstructed images. In comparison with the angular spectrum of GI reconstruction result, it is clearly seen that, as displayed in Fig. 4(e) and Fig. 4(f), the angular spectrum with more than 6 times wider can be retrieved by GISC. Further, generally speaking, the intensity values measured by the bucket detector *D_t_* satisfy a Gaussian distribution when the transverse size of the speckle illuminating the object is smaller than the object's dimensions^33^. However, for the case demonstrated in this paper, the bucket intensity values have a similar Rayleigh distribution (see Fig. 3(c)). By calculating the standard-deviation *δI* and the statistical mean 〈*I*〉 of the bucket intensity values, the ratio of its mean to standard deviation 〈*I*〉/*δI* = 1.16, which further validates the high-resolution ability of GISC. In addition, as the receiving areas of the detector *D_t_* are increased or the distance between the object and the detector *D_t_* is decreased, the quality of GISC will be improved (see Fig. 3(f) and Fig. 4(a–d)), which can be explained by Eqs. (5–7) (see Methods part) because the Euclidean term in Eq. (5) will approach zero such that Eq. (5) becomes the linear 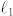-norm problem as the increase of the receiving system's numerical aperture (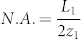)^23^,^24^,^32^.

**Figure 5:**
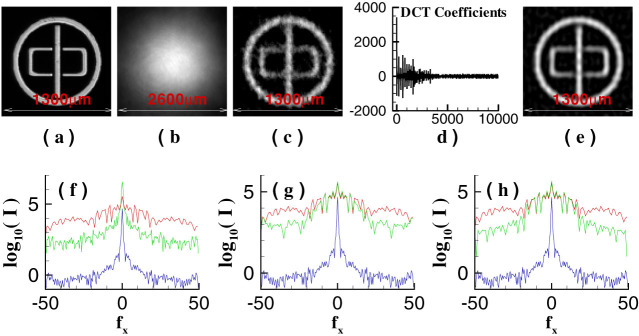
Recovered results of an aperture (“zhong” ring) in different representation basis, under the same conditions of Fig. 4 and *z*_1_ = 10 mm (*K* = 6000, namely 60% the Nyquist limit). (a). The object; (b). GI reconstruction; (c). GISC reconstruction when the object is represented in cartesian basis; (d). the object's DCT coefficients; and (e). GISC reconstruction when the object is represented in 2D-DCT basis. The green solid curves displayed in (f)–(h) are the cross-section of Fourier-transform distributions of the reconstruction results (b), (c), (e) at (f_x_, 0) direction, respectively. The red solid curve is the cross-section of Fourier-transform distribution of the object (a) at (f_x_, 0) direction. The blue solid curve is the cross-section of the image shown in Fig. 2(e) at (f_x_, 0) direction.

In order to verify the high-resolution ability of GISC for more general images and the effect of the object's sparse representation basis on the quality of GISC, as shown in Fig. 5(c,g) and Fig. 5(e,h), a transmission aperture (“zhong” ring, 100 × 100 pixels, the pixel size is 13 *μ*m × 13 *μ*m) is also reconstructed successfully by GISC when the aperture is sparsely expanded in cartesian and two-dimensional discrete cosine transform (2D-DCT) representation basis, respectively. It is clearly seen that the recovered image obtained in 2D-DCT representation basis is much better than that obtained in cartesian representation basis because the aperture has sparser representation in 2D-DCT basis, which means that using the same measurement data, the images with better quality can be achieved by choosing a proper representation basis^24^,^31^. Therefore, for the first time, we demonstrate experimentally that far-field high-resolution imaging can be realized by utilizing the object's sparsity constraint and random measurement even below the Nyquist limit in ghost imaging schemes.

## Discussion

By calculating the correlation function between two light fields, it is impossible for GI to obtain both the image in real-space of the double-slit and its diffraction pattern at the same time in fixed GI schemes^17^,^18^,^19^. However, by taking the image's sparsity as a priori, for far-field GI system shown in Fig. 1, when the speckle's transverse size at the object plane is much larger than center-to-center separation of the double-slit and the test detection plane is located in the far field of the double-slit, the double-slit's Fourier-transform diffraction pattern and its real-space image, as shown in Fig. 3(d,f), can be obtained by GISC method at the same time. Moreover, the reconstruction results of GISC don't only depend on how we measure the object as in GI frame (see Fig. 3(f) and Fig. 4(a–d)), but also depend on how sparse the object is in the representation basis (see Fig. 4(d) and Fig. 5(c,e)). Actually, for any GI system, we can find a suitable representation basis in which the object is sufficiently sparse, thus high-resolution imaging can be achieved and GISC will be a universal high-resolution imaging method. Understanding what happens at quantum level and the quantitative description of imaging resolution in GISC seem to be an interesting challenge deserving more investigation.

## Conclusion

In conclusion, by combining GI method with the object's sparsity constraint, we have achieved experimentally high-resolution far-field GI by using random measurement even below the Nyquist limit. Both the approaches to realize the linear 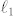-norm problem and an optimal representation basis can dramatically enhance the image's reconstruction quality. We have also shown that Fourier-transform diffraction pattern of the object and its image in real-space can be obtained by GISC method at the same time. This brand new far-field high-resolution imaging method will be very useful to microscopy in biology, material, medical sciences, and in the filed of remote sensing, etc.

## Methods

The intensity distribution 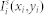 at the detection plane can be expressed as^8^
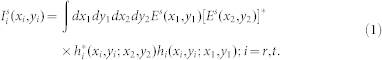
where the index *s* is defined as the *s*th measurement. *E^s^*(*x,y*) and [*E^s^*(*x,y*)]* denote the light field at the plane (*x*, *y*) and its phase conjugate, respectively. h_i_(*x*_i_, *y*_i_; *x*, *y*)(i = r,t) denote the impulse response functions of the reference and the test paths from the plane (*x*, *y*) to the plane (*x_i_*, *y_i_*).

### GI reconstruction

For ghost imaging^13^,^14^,^15^,^16^,^17^, the correlation function between the two detectors is:
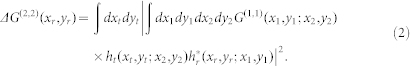
where *G*^(1,1)^(*X*_1_,*y*_1_; *X*_2_,*y*_2_) is the first-order correlation function at the source plane. By computing the intensity correlation between the intensity distributions 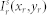 at the reference detection plane and the total intensities 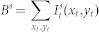 recorded by the detector *D_t_*, the object's image can be obtained without the utilization of the object's sparsity in the process of image restoration, namely called GI linear reconstruction algorithm^19^,^30^
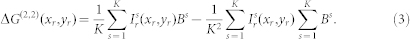
where *K* is the total measurement number. Using GI linear reconstruction algorithm described by Eq. (3), the results of GI with pseudo-thermal light demonstrated in Refs. 16, 17 suggest that the imaging resolution of GI is determined by the speckle's transverse size at the object plane (namely Δ*x_s_* ≈ *λz*/*D*). Therefore, for the scheme shown in Fig. 1, the object's image can not be resolved by GI linear reconstruction algorithm when the speckle's transverse size at the object plane is larger than the character of the object.

### GISC reconstruction

Mathematically speaking, any image can be expanded by an orthonormal basis (such as a Fourier basis and a wavelet basis). However, only a small number of the expansion coefficients are nonzero, and the largest coefficients can express the image's main features^23^,^24^. Therefore, the image is considered to be sparse or compressible in an appropriate representation basis, for example, a transmission double slit in Cartesian representation basis. Based on the theory of CS, there are an infinite number of images, which—after being convoluted by the random measurement matrix—will obtain the intensities recorded by the test detector for the setup shown in Fig. 1; our goal is to find the sparsest one. It has been mathematically and experimentally demonstrated that if the object is sparse enough, then any sparsity-based reconstruction method is bound to find the sparsest solution with measurements even below the Nyquist limit^22^,^23^,^24^,^25^,^26^. Employing the assumption of the object's sparsity in a representation basis, we try to realize far-field high-resolution imaging by using GISC method^30^,^31^. In the framework of GISC, each of the speckle intensity distributions 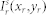 (*m* × *n* pixels) is reshaped as a row vector (1 × *N*, *N* = *m* × *n*) for GI system shown in Fig. 1. After *K* measurements, the random sensing matrix A (*K* × *N*) is reconstructed and meanwhile, the intensities (*B^s^*) recorded by the test detector *D_t_* are arranged as a column vector Y (*K* × 1). If we denote the unknown object as a *N*-dimensional column vector X (*N* × 1) and X can be represented as X = ψ·*α* such that *α* is sparse (namely there are only *K_c_* non-zero entries in the column vector *α*, *K_c_*≪*N* and ψ denotes the transform operator to the sparse basis), then the object X can be reconstructed by solving the following convex optimization program^32^:
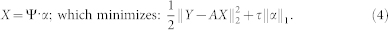
where *τ* is a nonnegative parameter, 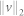 denotes the Euclidean norm of *V*, and 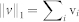 is the 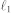-norm of *V*. Therefore, for the image with sparse cartesian representation, the reconstruction process of GISC shown in Fig. 1 can be written as follows based on Eq. (4):
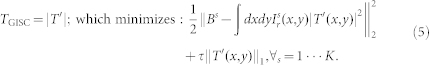
Where
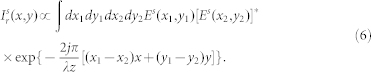

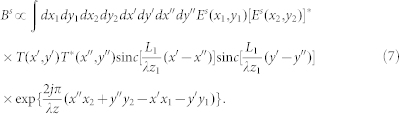


Here 
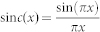
, *T*_GISC_ is the object's transmission function recovered by GISC method, and *L*_1_ is the effective receiving aperture of the test detector *D_t_*. Based on the theoretical analysis described in Refs. 22, 23, the imaging resolution of GISC will depend on both the object's sparsity in the representation basis and the mutual coherence of random measurement matrix. For GISC, in order to evaluate quantitatively the improvement degree of imaging resolution, we can measure the angular spectrum of reconstructed images compared with GI reconstruction result, similar to the k-space spectral analysis method^34^.

## Author Contributions

W.L.G. conceived the idea and provided the leading contribution to the experiments, collected and analyzed the data and wrote the manuscript. S.S.H assisted on the theoretical analysis and discussion. All authors contributed to the scientific discussion and revision of the article.
